# Exploration of Osteopathic Medical Students’ Reflections on Humanism in a Values-Centered Mentoring Program

**DOI:** 10.1007/s40670-025-02574-7

**Published:** 2026-02-03

**Authors:** Emily Young, Naomi Schmalz, Khadijah Guisse, Linda Regan

**Affiliations:** 1 Department of Clinical Affairs, Marian University Wood College of Osteopathic Medicine, 3200 Cold Spring Road, Indianapolis, IN USA; 2 Department of Medical Education, Creighton University School of Medicine, Omaha, NE USA; 3 Marian University Wood College of Osteopathic Medicine, Indianapolis, IN USA; 4 Johns Hopkins School of Medicine, Baltimore, MD USA

**Keywords:** Humanism in medicine, Mentoring, Reflection, Undergraduate medical education

## Abstract

**Purpose:**

Mentoring is common practice in professional development of future physicians and can contribute to developing values related to humanism in medicine. This study explores how diverse preclinical osteopathic medical students reflect on experiences in a mentoring program that focuses on humanism in medicine, related to values, settings, and contributors to their learning experiences.

**Method:**

The participants were first- and second-year medical students in one institution’s mentoring program from 2015–2023. The authors used a combination of inductive and deductive analysis to identify themes and subthemes in written reflections on humanistic values in response to learning experiences across 167 student-years. Purposeful sampling ensured inclusion of diverse participants. Saturation was achieved.

**Results:**

Preclinical osteopathic medical students reflected on all components of an established framework for humanism in medicine (integrity, excellence, collaboration, compassion, altruism, respect, resilience, empathy, service), as well as additional themes about assumptions/bias and holistic patient care. Participants’ reflections on humanistic values responded to learning experiences in both clinical and non-clinical settings across all themes. Diverse individuals (e.g. assigned mentors, other healthcare professionals, peer trainees, and patients and their families) inspired osteopathic preclinical students to reflect on humanistic patient care. Thematic nuances related to certain contributors and settings were identified.

**Conclusions:**

Learning experiences that involve engagement with diverse individuals in a range of clinical and non-clinical settings is a powerful way to prompt reflection on values related to humanistic patient care. This study expands our understanding of what humanistic patient care means to preclinical osteopathic medical students.

**Supplementary Information:**

The online version contains supplementary material available at 10.1007/s40670-025-02574-7.

## Introduction

Humanism in medicine is an important aspect of patient-centered medical care consisting of a set of professional values [1]. The Arnold P. Gold Foundation (APG) is a national organization that advocates, researches, and sets standards for humanistic patient care. They summarize humanism in medicine with the mnemonic, I.E.CARES, which stands for integrity, excellence, compassion and collaboration, altruism, respect and resilience, empathy, and service [2].

Mentoring relationships succeed when mentors and mentees connect over similar values [3], and mentoring can teach professional values [4]. Studies have identified mentoring as relevant for learner subpopulations like gender [5, 6] and racial/ethnic groups underrepresented in medicine [7–9], but without emphasizing specific humanistic values in those mentoring relationships. One study explored the role of one humanistic value (altruism) in persistence of learners underrepresented in their field, but did not focus on the role of mentoring [10].

Beyond interactions with mentors, students are affected by learning context such that a humanistic approach to patient care is shaped by environment, circumstances, and individuals involved in learning experiences [11, 12]. Layering reflection onto experiential learning catalyzes transformation [13]. In fact, mentoring with reflection is a common component of the professional development of physicians, particularly relevant to humanism in medicine [1, 14, 15].

While mentoring and reflection impact medical trainees’ professional development, little is published on its use to train medical students in humanistic values or on how context (individuals with whom learners interact and settings in which they learn) influences learning about humanistic values in medicine.

Marian University Wood College of Osteopathic Medicine (MU-WCOM) offers an extracurricular, non-credit-bearing, opt-in mentoring program for first- and second-year medical students. In this program, learners are assigned to healthcare professional mentors (e.g. physicians, hospital chaplains) for a span of one year; they are expected to meet with their mentors monthly throughout the academic year and reflect in writing on these active learning experiences. Students connect their written reflections to program values related to humanism in medicine as defined by APG [2]. This project studies MU-WCOM’s values-centered mentoring program to begin to address the gap in understanding how context shapes diverse mentees’ learning experiences related to humanism in medicine. The purpose of this study is to explore preclinical medical students’ reflections by investigating the following question: In a mentoring program focused on humanism in medicine for preclinical medical students, 1) what are diverse learners’ reflections on values, and 2) how are they shaped by a) individuals they encounter and b) settings in which their learning takes place?

## Materials and Methods

We combined inductive and deductive thematic analysis to explore preclinical medical students’ reflections in one values-centered mentoring program from 2015–2023. A previously published framework [2] defined the initial codebook, and we built and refined additional codes throughout analysis.

### Study Population

The study population consisted of preclinical medical students in MU-WCOM’s extracurricular mentoring program since 2015 (total enrollment ranging from 140–167 students/year). The data corpus included 1,060 student-years, out of 1,242 across eight years (approximately 85% of enrollees consented as research participants). A student-year refers to data from a learner who consented to use of their reflections for research during one program year. If that same learner re-enrolled and consented for a second year, they may represent two student-years in the data.

Students were invited to participate in the research at time of enrollment in the mentoring program from 2018-2023; students in the mentoring program prior to 2018 were retrospectively invited to participate in the research. Informed consent was obtained from all participants. See Online Resource 1 for sample informed consent language. IRB review was obtained.

### Data Collection and Sampling

Data consisted of learners’ written responses to a monthly assignment. The open-ended writing prompt invited reflection on program values and was consistent through the years, edited only for clarity of instruction. See Online Resource 2. Reflections were matched to learner attributes before de-identification and upload into qualitative analysis software, NVivo Version 14 (Lumivero, 2023). Students who did not consent to participate in the research were removed from the data before upload. 

We drew our sample from the data corpus with the goal of analyzing a diverse dataset. We purposefully sampled [16] by stratifying according to learner attributes, balancing traditionally under-represented with highly-represented participants as closely as possible. See Online Resource 3 for details of this process. In all, the sample we analyzed included 167 student-years. Table 1 shows how our sampling process represents the diversity of the data corpus within our data set.

**Table 1:** Attributes of participants in coded sample and data corpus (unit = student-years)

	Data Sample		Data Corpus	
Year	No	%	No	%
2015–16	20	12.0%	93	8.8%
2016–17	10	6.0%	112	10.6%
2017–18	13	7.8%	143	13.5%
2018–19	25	15.0%	157	14.8%
2019–20	25	15.0%	137	12.9%
2020–21	23	13.8%	140	13.2%
2021–22	27	16.2%	139	13.1%
2022–23	24	14.4%	139	13.1%
Total:	167	100.0%	1060	100.0%
Year in School				
OMS1	99	59.3%	667	62.9%
OMS2	68	40.7%	393	37.1%
Year in Program				
1st	111	66.5%	745	70.3%
2nd	56	33.5%	315	29.7%
Gender				
Female	90	53.9%	585	55.2%
Male	76	45.5%	374	35.3%
Nonbinary	1	0.6%	1	0.1%
not provided	0	0.0%	100	9.4%
Age				
20–29	132	79.0%	918	86.6%
30–39	30	18.0%	30	2.8%
40–49	3	1.8%	3	0.3%
not provided	2	1.2%	109	10.3%
SES				
Low	39	23.4%	123	11.6%
Middle	97	58.1%	620	58.5%
High	27	16.2%	206	19.4%
not provided	4	2.4%	111	10.5%
Race^a^				
American Indian or Alaska Native	6	3.6%	6	0.6%
Asian^b^	39	23.4%	157	14.8%
Black or African American	34	20.4%	34	3.2%
Hispanic^b^	27	16.1%	28	2.6%
Middle Eastern^b^	13	7.8%	13	1.2%
Native Hawaiian or Pacific Islander	8	4.8%	8	0.8%
White or Caucasian	75	44.9%	753	71.0%
Undefined^b^	1	0.6%	108	10.2%
Religion^a^				
Agnosticism	10	6.0%	87	8.2%
Atheism	11	6.6%	40	3.8%
Buddhism	4	2.4%	4	0.4%
Catholicism^c^	40	24.0%	283	26.7%
Hinduism	9	5.4%	46	4.3%
Islam	13	7.8%	36	3.4%
Judaism	7	4.2%	7	0.7%
Non-Catholic Christian^c^	46	27.5%	374	35.3%
Christian Orthodox^c^	7	4.2%	7	0.7%
Sikhism	11	6.6%	11	1.0%
Undefined^c^	13	7.8%	169	15.9%

This table shows the distribution of attributes of osteopathic medical student participants in the coded data sample compared to the data corpus. The data corpus includes all learners in a values-focused mentoring program at one institution between 2015–2023, who consented for their data to be used in research

Abbreviations: OMS1 = first-year osteopathic medical student, OMS2 = second-year OMS, SES = socioeconomic status

^a^ These demographic categories total > 100% due to participants selecting multiple identities

^b^ Some race/ethnicity write-in responses were combined into categories. *Asian* includes participants who selected the survey category Asian and participants who included the free text response Indian. *Hispanic* includes participants who selected Hispanic, Non-Caucasian Hispanic, and who included the free text response Cuban. *Middle-Eastern* includes participants who included free text responses: Afghan, Assyrian, Egyptian, Greek, Middle Eastern, Pakistani, and Persian (Britannica.com, [41])*. Undefined* includes participants who selected the survey category Other without an explanatory free text response and participants who left this survey item blank

^c^ Some religion write-in responses were combined into categories. *Catholicism* includes participants who selected the survey category Catholic and participants who included the free text responses Melkite Catholic and Roman Catholic. *Non-Catholic Christian* includes participants who selected that survey category and participants who included free text responses: Christian, Lutheran, Methodist, and Protestant. *Christian Orthodox* includes the following free test responses: Christian Orthodox, Coptic Orthodox, Greek Orthodox, and Orthodox. *Undefined* includes participants who selected the survey option “Other,” participants who left the survey item blank, and participants who included free text responses like the following: interested/believe in God but don’t follow a specific religion, exploring/figuring it out, not practicing, raised (a specific religion) but do not currently practice, and spiritual

### Thematic Analysis

Two coders (EY, NS) coded the sample. Inductive and deductive thematic analysis [17] of written reflections generated themes and subthemes related to program values as well as individuals and settings that students reflected upon. Coders worked individually over nine months, meeting approximately weekly. Coding meetings served to standardize our approach to applying the codebook, and iteratively develop and refine definitions, themes and subthemes, and reconcile discrepancies. We reached saturation after both coders had coded approximately 70% of the sample [18]; a single coder (EY) analyzed the remainder of the sample.

We began coding for the values outlined by the I.E. CARES framework using APG definitions where available [2]. We consulted Merriam-Webster’s online dictionary [19] for the two values without corresponding definitions (collaboration and resilience). We aimed to code each excerpt to a single, most representative value. We coded to a given value both 1) when a learner described it without explicitly naming that value in their reflection and 2) when a learner named the value they were reflecting upon.

Overlap of two or more values occurred in learners’ reflections (e.g. discussing empathy and compassion in the same sentence). In this case we coded the same passage to multiple values as a way of retaining context in support of the codes we chose.

We defined subthemes based on a combination of the content of learners’ reflections and additional published frameworks. Processes and decisions related to coding each individual value are elaborated within the results section.

### Reflexivity

Through this process, coders’ differing perspectives clarified rationale for coding decisions as well as relevance and significance of codes and how they shaped themes/subthemes. Coder 1 (EY) directed the mentoring program since 2015 and is trained as a physician. Her perspectives clarified the context of the data. Coder 2 (NS) had experience with qualitative methods in teaching and learning scholarship. Her perspectives supported initiation of software use and brought expertise to analysis. As an external reference point, Coder 2’s perspective increased trustworthiness [20].

## Results

**Fig. 1:**
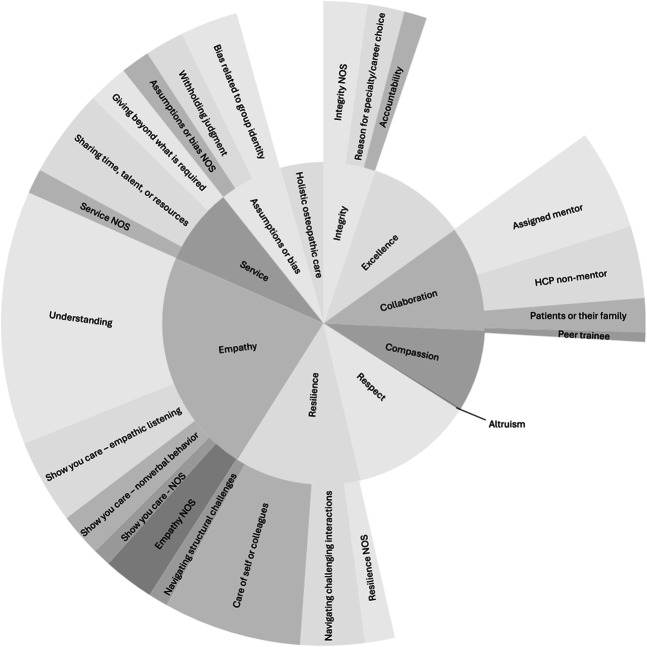
Relative frequency of themes (inner circle) and subthemes (outer circle) in osteopathic medical students’ written reflections on humanism in medicine in one values-centered mentoring program from 2015–2023. The shading differential of each theme/subtheme visually portrays the relative frequency of each. Abbreviation: NOS = not otherwise specified

Each I.E.CARES component [2] was a theme in the data set, and we identified two additional themes related to humanism in medicine. Themes varied in frequency with all represented strongly except one (altruism). Table 2 summarizes themes and subthemes, their definitions, and exemplar quotes. Figure 1 graphically displays the findings.

**Table 2:** Themes and subthemes in osteopathic medical students’ written reflections on humanistic values, with definitions and example quotations. The setting in which the learning activity took place and the individual who inspired the reflection are noted in parentheses after each example

Theme/Subtheme	Definition	Sample Quotation (setting, contributor)
Integrity NOS	“The congruence between expressed values and behaviors [2].” This theme captures learner reflections on lining up what they say or believe with what they do	“…without conscious and mindful effort we can drift towards behavior that doesn’t align with what we think is good. On a day that I’m stressed about exams or work, I might end up being quicker to judge the behavior of my patients or peers. However, if I have recently thought about my values of understanding, openness, service, and love, then my instinct might be to emulate those qualities instead.” (non-clinical, HCP non-mentor)
Accountability	Holding oneself accountable for one’s own actions and/or holding others accountable. E.g. owning up to a mistake, expressing concern about a negative role model, or admitting personal limitations	“…he emphasized the importance of being able to recognize when a mistake is made and then being able to admit to that mistake…. Our conversation made me think about all the qualities that I hope to possess as a future physician. I realized that being able to admit to when I am wrong is extremely important and is a standard I will be holding myself to in the future.” (non-clinical, assigned mentor)
Reason for career choice	Acknowledgment of a personal belief or feeling, in connection to a decision about specialty or career	“Medicine has been a resounding calling I began sensing as a young child…. I recognize and embrace that "to whom much is given, much is expected"…. And understanding this not only reinforces my deep respect for the profession but redoubles my motivation to make a significant contribution in it.” (non-clinical, assigned mentor)
Excellence	Expertise, as evidenced by clinical skill leading to positive patient outcomes or improved care. E.g. learner admires mentor’s procedural skills or medical knowledge	“While some of the procedures can be redundant (i.e. dermatological cancer excision), [my mentor] always reminds me of the different nuances present that make each operation a new experience. From the start he stresses the anatomical importance of every surgical procedure. Before you cut, you must know what is underneath. This not only ensures clinical excellence but in doing so also ensures the quality of work for the patient.” (clinical, assigned mentor)
Collaboration involving assigned mentor	Two or more individuals work ‘together, especially in an intellectual endeavor [19],’ involving the learner’s assigned mentor	“My day began observing her interact with allied health professionals and provide care to her patients. Through these observations I was able to see how impactful patient care can be when physician and allied health professionals such as nurses, social workers, physician assistants, and therapist work cohesively.” (clinical, assigned mentor, HCP non-mentor)
…involving another HCP	Collaboration involving a HCP who is not the learner’s mentor. E.g. learner reflects on importance of teamwork between two or more HCPs	“He is a fantastic model of what all providers taking care of patients should be, and sets an excellent example of the importance of both team work and humanity in medicine.” (clinical, HCP non-mentor)
…involving a patient or their family	Collaboration involving a patient or patient’s family. E.g. shared decision making or a patient or their family member contributes to problem-solving to overcome barriers to medical adherence	“He said it is heartwarming to have these conversations that allow him to build such a special relationship with his patients, where they form a partnership and work together as a team rather than one instructing the other what to do. He described how in this partnership there are two experts: [Dr. X] being the expert in oncology and the patient being an expert in themselves….” (non-clinical, patient or their family, assigned mentor)
…involving a peer trainee	Collaboration involving another trainee (e.g. medical student of any level or learner in another discipline) other than the participant	“With another student, we took the patient history and did a physical exam prior to presenting the information gathered to the physician.” (clinical-safety net clinic, peer trainee, HCP non-mentor)
Compassion	“The awareness and acknowledgement of the suffering of another and the desire to relieve it [2].”	“I witnessed a physician and nurse talking to the family of a young patient regarding organ donation. The family was emotional and both the physician and nurse were doing their best to console them, including sitting and holding hands and giving hugs.” (clinical, assigned mentor, HCP non-mentor)
Altruism	“The capacity to put the needs and interests of another before your own [2],” to the point of self-sacrifice that benefits another more than expected by one’s professional role [25]	“Humanism was seen through our team members sharing their only sandwiches with the interpreters working with us that didn’t have anything to eat…” (clinical, HCP non-mentor)
Resilience – NOS	“Ability to recover from or adjust easily to misfortune or change [19].” E.g. getting through difficulties	“[My mentor] had mentioned that a "well rounded person" isn’t someone who necessarily gets a 4.0 and is a part of a few organizations. We agreed that it would be someone who can talk to people, lead a team and be calm under pressure.” (non-clinical, assigned mentor)
Care of self (or colleagues)	Positive coping strategies to avoid burnout, like self-awareness, realistic expectations, work-life balance, participating in a support system, or finding purpose in one’s work	“As such I need to find ways to prevent myself from burning out, such as exercising regularly and being an advocate for my fellow physicians by supporting measures that could help make medicine a more positive environment for everyone involved, which in the end ultimately goes to serve patients by ensuring that their physicians are in a good place to treat them.” (non-clinical/online module)
Navigating challenging interactions	Ability to respond effectively to ‘difficult’ patient scenarios or team dynamics. E.g. responding to strong emotion	“She also spoke to me about how to interact with patients who get angry or express strong negative emotions. She tries to put herself in the shoes of her patients, and it helps her to remain calm and try to deescalate the emotional state of her patients. I will use this advice as a practicing physician when I encounter patients who may be struggling emotionally.” (non-clinical, assigned mentor)
Navigating structural challenges	Ability to adjust to systemic limitations, e.g. institutional or insurance barriers to care	“I admired how [Dr. X] interacted with each patient with a calm demeanor although she was very stressed about being behind…. It is unfortunate that annual check-ins have been reduced to only 10 min with the patient, which seems impossible on a day-to-day basis. Nevertheless, [Dr. X] managed to ask each patient something personal about their life that she remembered from previous visits.” (clinical, HCP non-mentor)
Respect	“Regard for the autonomy and values of another person [2].”	“Another important thing is properly informing the patient of what procedure will be done and why, and of course, asking their consent.” (clinical, assigned mentor, HCP non-mentor)
Empathy – NOS	The ability to put oneself in another’s situation [2]	“I think that learning to empathize with those we have very little in common with is a vital contribution to a humanistic approach to patient care. I would like to expose myself to as many different cultures and ideas as possible, so that I may better relate to a variety of patients.” (non-clinical/online module)
Show you care – NOS	Learner describes empathy with response that demonstrates care or states someone showed they cared but does not elaborate detail	“Just how patients want to go to doctors office where the physicians actually care for the patient, physicians want to work for a hospital that cares for them.” (clinical, assigned mentor, HCP non-mentor)
Show you care—Nonverbals	Nonverbal behaviors that communicate concern, interest, or connection, e.g. eye contact, warm tone of voice, body posture [22]	“…[a pediatric patient] was all by himself, the hospital was unable to contact his parents…. the doctors and nurses on the floor took time to visit and talk to [him]. He might not be able to understand but he can feel the presence of people who really care for him.” (clinical, assigned mentor, HCP non-mentor)
Show you care – Empathic listening	Forms of speech that enable another to know their perspective is welcome, e.g. use of silence or prompts that validate, explore, and/or ‘signal it is ok to express feelings [21].’	“[Dr. X] took time to address multiple concerns that the patient seemed to be adding as the exam progressed, making sure the patient knew that she was being heard and that she was the priority at that moment.” (clinical, assigned mentor)
Understanding	Ability or efforts to see or experience another’s perspective or feelings [21], e.g. HCP comes to understand patient perspective through experience (HCP as patient [2])	“This is something I will value a great deal in practice and can appreciate as someone who has needed an anesthesiologist before. The moments right before you let someone breathe for you can be very frightening, especially for a child or someone who has little experience with invasive procedures.” (non-clinical, HCP non-mentor)
Service – NOS	Learner wrote about providing healthcare as a service, without elaborating	“In all of its history, the physician has always [been] in the position of servitude and if there is anything that ensures that the sick (or patient) is always the focus, it is critical for future physicians to embrace this view.” (non-clinical/online module)
Sharing time, talent, or resources	Learner’s reflection focused on patient care as sharing of resources, including time or talent, usually with someone in need [2]	“One thing I noticed is that [Dr. X] always took time during the visits to explain things to her patients. Whether that be interpreting lab work in a way that the patient could understand, or printing out articles or handouts for them to read. In a way I found this to be an example of humanism. It could be easy to rush through visits given her busy schedule, but she always took time to make sure the patient understood what was going on.” (clinical, assigned mentor)
Giving beyond what is required	Learner offers the perspective that someone went ‘above and beyond’ or did more than expected of them, as an act of service [2]	“What stood out to me this time was how [Dr. X] went above and beyond to advocate for her patient. For example, one of her patients was from Mexico, and was having trouble figuring out how to get her husband a green card. [Dr. X] scheduled an entire appointment with her so she could help understand and get things figured out. She ended up writing a letter for her, discussing her medical history, that would hopefully help in the process. I felt that this was a good example of humanism, because [Dr. X] went beyond just medicine to help her patient.” (clinical, assigned mentor)
Assumptions or Bias – NOS	Learner comments on identifying and managing assumptions or bias as a value in patient-centered care	“Even though we may think patients are going to give us difficulties, the most important thing to focus on is their care.” (clinical, assigned mentor)
Bias related to personal identity	Learner reflects on assumptions or bias related to someone’s identity, e.g. age, gender, race, ethnicity, socioeconomic status, or profession	“A clinician…expressed that in her experiences she witnessed inconsistencies in addressing patients in different health settings and with certain patient populations. For instance, in certain health care settings that are more accessible to individuals with a low socioeconomic status, health care providers may refrain from using titles such as Mr., Ms. Dr. and instead will address a patient by the first name upon their first interactions; Rather than initially addressing the patient by their title and then asking what the patient would like to be addressed by. The clinician noted that in hospitals that were in nicer areas and had updated equipment many healthcare providers will uphold professionalism and respect when addressing the patients in those settings.” (non-clinical, assigned mentor, HCP non-mentor)
Withholding judgment	Learners reflected on the value of not judging a patient, peer, their mentor, another HCP, etc	“I believe attending the AA meeting will help me to treat all patients with respect, and to never judge a patient’s situation because you never know what they have been through in their life.” (non-clinical, patient or their family)
Holistic, osteopathic care	Treating the patient as a whole, E.g. a ‘mind–body-spirit’ mindset or addressing spiritual needs in addition to physical	“[Dr. X] and I talked about what defines a highly humanistic physician. We both agree that the osteopathic tenet of “the body is a unit” is a key foundational component. Also, a physician needs to be able to look past the disease and treatment of the disease and consider other determinants of health as well. Too often determinants of health are an upstream cause contributing to disease and they can be addressed if given appropriate attention.” (non-clinical, assigned mentor)

Abbreviations: AA = Alcoholics Anonymous, HCP = healthcare professional, NOS = not otherwise specified

### Setting and Contributors

Across all themes, learners wrote about values based on experiences in both clinical and non-clinical settings. Individuals in many roles inspired written reflections–not only assigned mentors, but also other HCPs (including resident physicians and healthcare administrators), patients or their family members, and peer trainees (other medical students or trainees from other programs). Assigned mentor-student pairs sometimes developed their own longitudinal focus across multiple meetings.

### Themes and Subthemes Related to Humanism

#### Integrity

As with other themes, we began coding for integrity using APG’s definition [2]. Two nuances became subthemes. Learners discussed the importance of physician *accountability* for their actions, for example by admitting personal limitations, e.g.: “…the more humanistic approach is to be honest and tell the patient that we are not sure what is going on with them, but these are the steps we will be taking to figure it out….” Students also connected with mentors over *making career decisions based on personal beliefs or values*; one student discussed observations with her emergency physician mentor about how specialty training equips physicians differently for addressing public emergencies: “I liked that [my mentor] shared with me this story because this is exactly one of the reasons why I wanted to pursue EM….the field is broad and allows me to help people with most of their medical issues….”

#### Excellence

We clarified APGs definition of *excellence*, “clinical expertise [2],” to specify procedural or cognitive clinical skills, as opposed to expertise in other humanistic skills. In clinical settings, students observed HCP’s behaviors that they associated with excellence (good patient outcomes), like performing thorough patient interviews and examinations, having and applying medical knowledge, and educating patients. As written by one student who observed her mentor administer in-office treatments to patients with complex needs: “… I realized how important it is to be a good listener, knowledgeable, and skillful.” Another praised her mentor for “efficiently conducting a clinic visit.” A third identified excellence in non-mentor HCPs: “the nurses there took great care in training the patient’s parents on placing the NG tube, making sure to answer any and all questions they had.” Non-clinical settings also inspired reflections on excellence, as illustrated by a learner’s report that their mentor emphasized, “…it is always important and necessary as a physician to keep learning and refreshing old knowledge… so that we can be the very best versions of ourselves for our patients.” In these ways, experiences in clinical and non-clinical settings, with assigned mentors and other HCPs, contributed to students’ reflections on excellence in patient care.

#### Collaboration

We defined *collaboration* as “two or more individuals working together, especially in an intellectual endeavor [19].” Subthemes naturally formed around categories of individuals who contributed to collaborations: *assigned mentor, non-mentor HCPs*, *patients or their family*, and *peer trainees*. When students wrote about collaboration, they described collaboration between members of the healthcare team (often including their assigned mentor), how it was done well or why it was important, for example, “…seek(ing) out others for help will show my colleagues that I respect their opinions and it will show my patients that I am seeking other’s opinions to ensure they receive the best possible treatment.” Learners described physician–patient collaborations they observed–”[My mentor made] the patient an equally important partner in her own healthcare….”– and how their mentors discussed with them their work with patients–”[My mentor] told me that his goal…(is) to guide the patient with his advice so that they can do…what they believe is the best option….”

While learners discussed peers less frequently, we found the presence of peers as collaborators to be meaningful. Peer collaboration came up in particular settings, like shifts in a student-run safety-net clinic, where students “collaborate(d) with each volunteering team, including the supervisors, team aides, and translators.” Collaboration with other trainees also arose in clinical simulations involving nursing students and medical students, described as a “comfortable setting to give feedback on the relationship and communication needs of both parties.” These reflections illustrate collaboration by multiple contributors and emphasize its value in both clinical and non-clinical learning settings.

#### Compassion

The APG definition of compassion focuses on awareness of another person’s suffering, coupled with a desire to relieve it [2]. This theme carries the idea of responsive action, or at least the desire to act. Students wrote about physicians advocating for change, represented in this quote about a mentor: “she practices medicine because she is passionate about helping women who are suffering.” Others described learning about compassionate patient-physician interactions from patients themselves, like a patient who described that when “he started having a reaction to a medication in the infusion center…[Dr. X] was down by his side before even the main nurse could get to him. I think that is the type of care and compassion a physician should have….”

While *compassion’s* definition remained straightforward, we found it helpful to compare and contrast with similar values (e.g. empathy and service) as they developed subthemes. For example, compassion is specific to suffering, while empathy responds to another’s emotion of any kind. To tease out compassion from altruism, we considered who was benefiting vs. who was inconvenienced in the scenario. Compassion did not necessarily involve personal sacrifice, whereas altruism did.

#### Altruism

*Altruism* was notable for its low frequency in the data. Based on APG’s definition, our coding searched for ideas of self-sacrifice and personal inconvenience [2]. In 167 student-years, participants used the term altruism/altruistic only twice, and our manual coding expanded the total number of reflections on altruism to fewer than ten. One reflection described the altruism of a HCP in the clinical setting who voluntarily inconvenienced herself beyond what was expected of her professional role, for the benefit of the learner. The student said, “I practiced starting an IV on the nurse. It was an interesting experience because the first time I did it the nurse bled everywhere but she was kind enough to let me try on her other arm.” While altruism appeared infrequently in our data, the emphasis on prioritizing resilience was prominent.

#### Resilience

Coding for resilience began with Merriam-Webster’s definition, “ability to recover from or adjust easily to misfortune or change [19],” and developed four subthemes; *care for self or colleagues, navigating interpersonal challenges*, *navigating structural challenges,* and *resilience NOS*. Most reflections responded to conversations with or direct observation of learners’ assigned mentors. Reflections inspired by non-mentor HCPs were similar in content and meaning.

The subtheme *care of self or colleagues* featured conversations with and observations of mentors and other HCPs about physician health and burnout, stigmas that inform physician health behaviors, and creating/maintaining work-life balance. A student illustrating this subtheme described a discussion with their mentor and mentor’s spouse (also a physician), saying, “[they] shared their personal study habits and how they set time apart during the weekends to dedicate to socialization/recreational events. They emphasized the importance of setting that time for yourself to prevent burn out….” Students also reflected on patients’ and their families’ contributions to the importance of and strategies for self-care. In clinical settings, students were inspired by patients’ resilience in the face of challenging health events. For example: “In the face of so much adversity and uncertainty, this patient…had every right to be frustrated and upset at what had occurred, but instead she chose to be grateful…. I hope to be as resilient and positive as her….” Students who reflected on time spent in non-clinical settings with patients and peers, commented on the importance of community for personal and professional wellness, as in this example from a patient support group: “One thing I took away from this AA meeting is the importance of having a support system, not only from your family and friends, but also from the community, especially from people who share similar stories and struggles….” In sessions where learners discussed life experiences with peers, they commented on perspective gained by listening to the struggles of others different from their own, for example: “stories like [my peer’s] put things into perspective and help me remember that even though medical school has been and will continue to be a journey full of hard work…it is a privilege and honor to be in it.” Thus, various settings and contributors inspired learners to reflect on self-care as a coping mechanism, including forming relationships with colleagues for bidirectional support.

Another resilience subtheme focused on *navigating challenging interpersonal scenarios*, or responding in a positive way to difficult interactions in the clinical setting. Students often cited discussions with their mentors as a source of wisdom within this subtheme, whether about managing a patient’s addiction, non-adherence, or complex problem list or handling someone else’s emotion. One student wrote:

I will have to deal with people that may be unpleasant,...upset…[or] angry at me for mistakes I made. I need to face that reality and learn to embrace these interactions instead of fearing them,….deal with unpleasant situations with grace, with eagerness to learn and willingness to improve myself, and with the ability to deflect off nasty comments or actions….

While this subtheme includes resilience related to interpersonal exchanges, a final resilience subtheme addresses the macro- level: *navigating structural challenges*.

Reflections on structural challenges described the importance of resilience while working within a system that places limits not only on one’s ability to practice in a preferred way (e.g. through productivity demands or insurance bureaucracy), but also on patients’ limited access to resources important to their health. For example:

Being able to have access to food and facilities that will allow people the opportunity to change their habits [is] very needed. Working out when you are stressed may be considered a privilege, not a right, to people who work multiple jobs to afford basic needs, have children who must be cared for, or simply don’t have gyms in their area. From this experience, I learned how important it is to be aware of the community you are serving.

#### Respect

*Respect* is “the regard for the autonomy and values of another person [2].” Based on a learning experience in a patient support group, one learner reflected, “…every patient comes in with their own story, values, priorities, and current station in life, regardless of the specific medical condition that brought them to clinic.” Learners also discussed respect in its relevance to patient choice in medical decisions, observing that a humanistic clinician, “knows what the specific goal of [a] patient is and uses [the provider’s] unique style to help them accomplish that goal.” These reflections emphasized that respect in clinical care includes recognizing each patient’s individuality and supporting their autonomy.

#### Empathy

APG’s definition of empathy, with its primary focus on perspective-taking [2], started our analysis. However, we sought additional input from the literature to refine coding, which also led to subthemes. Agosta talked about openness to another individual’s emotion, understanding, and active listening as components of empathy [21]. Another empathic habits framework overlapped with Agosta’s description and added nonverbal behaviors [22]. Recoding our sample in light of these published perspectives surfaced five empathy subthemes: *empathy NOS, show you care (SYC)-NOS, SYC-empathic listening, SYC-nonverbal behaviors,* and *understanding*. Frequently learners reflected on multiple empathy subthemes, moving from one to the next and back, as illustrated by this learner, who perceived that a hospitalized patient wanted some company:

*I took the time to just talk with him about random things*, and also helped him get his glasses and move a flower vase over to his bedside so that they were closer to him. All of **these very simple tasks seem to really make a difference to this patient and bring him a lot of joy…**.

(subthemes demarcated by italics, underline, and bold font: *SYC-empathic listening*, SYC-nonverbal behavior, **understanding**). Here, the *understanding* subtheme showed a learner recognizing another person’s emotion (joy). *Understanding* also captured emotion a learner internalized from another individual, like reflection on “…heart-breaking moments, of witnessing my patient’s death [and the family’s grief]…” and instances where a learner attempted but felt they could not accurately perceive the depth of a patient’s emotion, as in, “I cannot imagine how difficult it is to go through what she does on a daily basis, knowing that there is no cure….” That is, empathic understanding not only meant an ability to experience someone else’s emotion along with them but also an ability to recognize when another’s life experience was so profound that it remained beyond comprehension.

#### Service

APGs bipartite definition for service shaped the subthemes [2]. Learners’ reflections that discussed *sharing time, talent, and resources with those in need* often focused on caring for patients or populations who were underserved in some way. Many of these excerpts were inspired by a learning experience in a safety net clinic or a clinic whose mission related to healthcare access, as this learner described:

[My mentor] …opened this facility…after a year of planning and researching to find the right building, area, and community…. His business provides medical care to an underserved population in the middle of [the city]. A visitor to the open house told me that she was excited about the urgent care clinic because, before it opened, she had to take her kids to [a different city] for urgent care. [My mentor’s] clinic was much closer, more accessible, and more attuned to the local community’s medical and financial needs.

Students also wrote about observing, discussing, and/or acting out the subtheme *giving beyond what is required*. In one example, a student described his efforts to tend to a patient apart from routine history taking and physical examination:

The boy…never said anything,...but he smiled when I tried to make him laugh…. I got him to open his mouth so I could see his oral cavity and then palpated his swollen neck. The most important things I did for him though, were to compliment him on his Teenage Mutant Ninja Turtles sweatshirt… and lift him on and off the exam table.

### Themes and Subthemes Outside the Existing Framework

Written reflections manifested two strong patterns that did not fit clearly within I.E.CARES [2]. Both developed early in coding.

#### Assumptions and bias

Learners discussed the interface of assumptions and bias with patient care. The subtheme *bias related to group identity* encompassed HCP and patient identities like gender, race, parental status, and profession (including allopathic vs. osteopathic credentials). Students also discussed biases impacting patient care based on labels or pre-existing conditions, as in, “a bias was established towards this patient based on his addiction …[and as a result] he might have died of septic shock.” Another discussed “preconceived notions…[related to patients’] cultural and socioeconomic backgrounds.” *Withholding judgment* formed another subtheme*.* This learner discussed the importance of a physician avoiding judgments about patients, in response to an open Alcoholics Anonymous (AA) meeting he observed:

…there was a gentleman at the meeting, about my age…. if I would have seen him on the street I wouldn’t have even taken a second glance…. Without even consciously noticing it I had already judged…. Although he only spoke briefly I got a feeling that he was going through a lot more personal hardships than I had initially assumed. Just from hearing him talk I realized how easily I could be this guy…I think trying to deduce a person’s problems from their appearance is nothing more than a recipe for disaster especially in terms of providing humanistic patient care.

#### Holistic Osteopathic Care

A final theme from the data reflected an aspect of participants’ foundational professional philosophy, which teaches that a patient is a unified “body, mind and spirit [23].” Consistent with this principle, learners frequently wrote about “…the holistic approach we aim to achieve. Practicing the principle of treating the mind, body, and spirit…” Finding this theme in our results shows that osteopathic medical students see this foundational tenet of their future profession to be an important part of patient-centered, humanistic care.

## Discussion

This study shows that medical students in a values-centered mentoring program reflect across the range of humanism in medicine as previously defined in the I.E.CARES framework [2]. In addition to the widely accepted framework for humanistic patient care, learners raised issues about assumptions/bias and holistic osteopathic care, as relevant to humanism in medicine. Both clinical and non-clinical settings inspired reflection on values across all themes. That said, we found a handful of nuances related to how students reflected on humanism in response to learning experiences with certain settings and individuals. Our findings can inform the use of experiential learning strategies to develop humanistic qualities in medical trainees.

### Key Findings Related to Existing Humanism Framework

Our learners applied the gamut of APG’s framework [2] to patient care as they developed and reinforced their own values related to humanism. Among the I.E.CARES [2] components, altruism’s relatively low representation in students’ writing supports prior assertions that the culture of Western medicine has shifted away from valuing physician self-sacrifice [24]. Consistent with our findings, Glannon and Ross comment that true altruism is rare in contemporary medicine (in contrast to beneficence, an expectation of physicians) in that it involves the exchange of more than what is expected in a patient-physician relationship [25]. Another perspective on altruism’s low representation in our data could cite the variation in published definitions for altruism [2, 25–27]. For coding purposes, we used a narrow operational definition in order to distinguish altruism from other values. While possible that a restricted definition alone could reduce frequency of a qualitative theme, this explanation does not account for other claims in medical education literature, described above [24, 25]. Regardless of the reason, these findings suggest that instructors who wish to develop altruism in their learners should provide explicit teaching on definitions and examples.

### Key Findings Related to Settings and Individuals

Our findings suggest that learners’ reflections on humanistic values can be inspired by *any* individual or learning setting. Still, we identified a limited number of nuances related to specific contributors and settings in our cohort.

#### Patterns Related to Setting Included Nuances in Institutional, Non-clinical, and Clinical Learning Settings

Regarding settings that inspired learners’ reflections on humanism, we noted three patterns. First, we found the theme of holistic care related to the osteopathic training setting in which this mentoring program is administered. While we anticipate similar themes may surface in other settings, it is not surprising that this pattern presented strongly at this particular institution, given its foundational osteopathic tenet of mind–body-spirit integration [23]. Second, while all our themes appeared across both clinical and non-clinical settings, we found the theme of assumptions and bias was notably impacted by learning experiences in non-clinical settings, especially patient support groups. Learners discussed two assumptions/bias subthemes (*bias due to group identity* and *withholding judgment*) in response to observations at 12-step program meetings. The prominence of our withholding judgment subtheme, in particular, aligns with prior suggestions that AA attendance can serve as a learning tool to help mitigate stigma among future physicians [28]. Finally, we noted that the safety-net clinic setting prompted reflection on a specific aspect of service: sharing time, talent, and resources with underserved patient populations. Similarly, Smith and colleagues found that community service in the form of student-run clinics, followed by reflection, may positively shape future physicians’ attitudes toward underserved patients [29]. Other researchers have suggested that working in student-run free clinics may protect against development of negative attitudes about caring for patient populations that are underserved, though their intervention did not leverage reflection as a learning tool [30].

#### Patterns Related to Contributor + Setting Clustered Around Two Particular Values: Collaboration and Resilience

Collaboration and resilience surfaced as two specific values with nuances related to contributors and settings. Whereas learners reflected on collaboration between mentors and other HCPs as teamwork, their descriptions of collaboration between patients and HCPs often focused on aspects of shared decision making (SDM). Studies on teaching preclinical medical students about SDM are rare [31, 32], and our findings suggest that providing opportunities to observe and reflect on SDM could introduce medical students to these skills early in training. Peers contributed to reflection on collaboration in student-driven settings like interprofessional simulation activities and student-run safety net clinics. Other scholars identified similar findings. Huang and colleagues investigated collaborative learning among multidisciplinary peer trainees in the setting of student-run clinics, including the learning setting’s impact on patient-centered care for marginalized groups [33]. Carpenter and colleagues found value in using simulated clinical activities to develop collaborative skills between nursing and osteopathic trainees [34].

Patients contributed to reflection on resilience in support group settings. AA support groups featured prominently in our preclinical learners’ reflections, while other studies explored third-year students’ clerkship experiences of AA meetings [28, 35, 36]. Kastenholz and Agarwal’s analysis of medical student reflections on AA meetings noted a theme of spirituality and hypothesized a connection between this aspect of AA and resilience [35]. Balasanova and colleagues found third-year students’ observations of AA meetings to build humanistic perspective by mitigating stigma [28], but did not highlight resilience in AA attendees like our learners did. In response to both patients in support groups and peer trainees in multiple settings, our learners reflected on resilience that comes from a sense of community based around listening to or identifying with others’ challenges. They expressed comfort from knowing peers’ challenges were similar to their own and perspective gained from hearing others’ struggles different from their own, not unlike ways other health professions students experienced support from peer communities [37]. Community support in our findings also is reminiscent, on a small scale, of cohort-based programs that develop medical student resilience [38]. A final, strong contributor group to reflections on resilience included mentors and other HCPs. Participants reflected on health professional role models and advisors in ways related to physician health and burnout, stigmas that inform physicians’ health behaviors, and creating/maintaining work-life balance. Our resilience subthemes are consistent with other models suggesting the relevance of role modeling and mentoring from advanced professionals to medical trainee resilience [39, 40]; however, our findings expand earlier thought by elaborating nuances in how trainees may reflect on resilience in response to different contributors.

## Summary of Discussion of Key Findings

We have discussed patterns in diverse learners’ reflections on humanistic values and how context (setting and individuals) shape them. Our study affirms prior work showing that the ability to reflect on experiential learning, especially when facilitated by mentors and role models, supports developing humanistic professional values [15]. It also expands our understanding of what humanistic patient care means to osteopathic medical trainees, including emphases on 1) recognizing and mitigating assumptions and bias in healthcare, and 2) holistic osteopathic patient care. Finally, we have presented nuances related to certain settings and contributors to learners’ reflection on values, which affirm and expand upon related studies.

### Strengths and Limitations

Study design strengthens our validity evidence. Data pulled from a large data corpus (1,060 student-years) creates a robust sample. Using data from regular educational processes adds trustworthiness because timely reflections represent learner experiences more credibly [20] than data gathered later. Sample stratification by demographics improves transferability of qualitative findings to diverse contexts [20]. Other researchers explored development of humanistic values without stratifying their qualitative sample to ensure diversity [15, 34, 36], or they investigated perspectives of underrepresented/minoritized participants apart from well-represented ones [5, 7, 9], in line with their research questions. Our research question allowed us to use a sampling strategy aimed at balancing underrepresented and well-represented participants across the data set.

This study focused on osteopathic students at a single medical school. The transferability of these findings to allopathic settings remains unknown, especially given the relevance of one of our thematic findings to osteopathic tenets [23]. This study also was not designed to confirm nuances based on participant identities: the open-ended prompt allowed learners to focus on what they found most salient, whereas a more targeted prompt might have yielded different perspectives by identity. Additionally, because learners opt-into the mentoring program, the study participants may differ from those who did not enroll in ways that relate to personal characteristics and baseline values. This may also affect transferability of findings to other learners.

Another potential limitation arose from a need to create operational definitions for purposes of coding certain values into discrete categories. Sometimes, our definitions created boundaries that felt artificial given the nuanced–even controversial–perspectives on how certain values (e.g. compassion, altruism, empathy, and service) mean different things to different people [2, 21, 22, 26, 27]. A more targeted writing prompt about how these concepts manifest in clinical settings might have better elucidated how these values impact patient care. That said, while discrete definitions matter for coding, they may not make a difference in how values play out in patient-physician relationships. For patients and colleagues, naming which values are at play is not important as long as they are present.

Future work could explore further what students learn about specific humanistic values in specific settings (e.g. patient support groups) or with specific individuals (e.g. hospital chaplains), or explore similar interventions in an allopathic training setting.

## Implications

Exposing students to humanism in medicine through reflection on experiential learning in clinical and non-clinical settings meaningfully supplements classroom activities. There is great diversity in learners’ reflections on values and how they are influenced by learning context. That is, students learn about humanistic values in medicine by not only engaging with assigned mentors aware of the program’s focus on values but also observing and interacting with individuals other than their mentors. Additionally, experiential learning in clinical and non-clinical settings is a powerful way to prompt meaningful reflection on values related to humanism in medicine.

In particular, reflection on values may be one way for osteopathic medical schools to assess the discipline-specific identity formation of their learners. Medical schools, in general, could leverage experiential learning in certain settings, like patient support groups and safety-net clinics, to contribute to learning outcomes about values like resilience, collaboration, and assumptions or bias. For the mentoring program we studied, these findings encourage review of written reflection prompts to inform potential revision. Less open-ended, more targeted prompts may allow faculty in this and other mentoring programs to guide learner reflections toward specific values, for example those that are mission-aligned yet underrepresented in learning outcomes (like altruism). Instructors can also word reflection prompts to specify particular values for learners to reflect upon in response to specific settings or individuals (e.g. collaboration in response to interactions with HCPs or peer learners, service in the setting of safety-net clinics).

## Supplementary Information

Below is the link to the electronic supplementary material.

Supplementary file1 (DOCX 247 KB)

## Data Availability

Raw data are not publicly available, in order to preserve individual participants’ privacy according to the informed consent agreement.

## Declarations

### Previous presentations

The authors presented partial, preliminary results as a work-in-progress at the International Health Professions Education Virtual Conference on October 18, 2024, titled *Exploration of Medical Students’ Reflections in a Values-Centered Mentoring Program*.
