# Supplementary material for: Exploration of Osteopathic Medical Students’ Reflections on Humanism in a Values-Centered Mentoring Program
Source: Med Sci Educ. 2026 Feb 3;36(2):939–53. doi: 10.1007/s40670-025-02574-7 (PMC13197506; doi:10.1007/s40670-025-02574-7)
Supplement: Supplementary file 1 — Supplementary file1 (DOCX 247 KB) [file 40670_2025_2574_MOESM1_ESM.docx]

**Online Resource 1**

*The text below consists of sample informed consent language used to invite learners enrolling in the mentoring program to participate in this research study.*

The **primary purpose of this survey is to gather information from you that will help us match you to an appropriate mentor in the Meaningful Medicine Mentoring Program (MMmp)**.

A **second purpose of this survey is to request your informed consent to let us include your written reflections in this program in MMmp-related research (IRB #21.291)**.  We plan to review students’ de-identified reflections for general themes on what students learn about humanism and how they experience mentoring through MMmp.

In addition to the usual review of your reflections by program directors and teaching assistants, if you consent to participate in the MMmp-related research, investigators (e.g. Dr. Young) and research assistants may also analyze your reflections. Your reflections will be de-identified and then analyzed as a large mass of students’ written comments (a large group of text). Your personal identifying information will not be included in our data or publications.  Although we cannot promise complete anonymity (e.g. because Dr. Young, a co-investigator, gets to know students in the MMmp), we will uphold your confidentiality.  This means that during our data analysis, even if a researcher is able to identify who wrote a reflection, they will not name or reveal the identity of the person in the results.

Declining to participate in MMmp-related research carries **no penalty** and does not disqualify you from enrolling in MMmp.  Furthermore, you can change your decision later in the year by notifying Dr. Young in writing.

The only required question below is whether you are willing to accept the conditions of the Teaching Assistant role in this program (the final question), which is independent of your consent to let us use your reflections in our research.  The other survey questions are voluntary.  However, the more information you can provide, the more it will help us to match you to an appropriate mentor.

You may contact a co-investigator for the MMmp-related research if you have any questions about the research (Dr. Emily Young: eyoung@marian.edu or 317-955-6627).


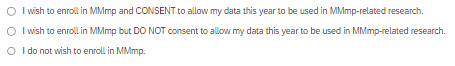


**Online Resource 2**

*The assignment prompt below is an example of the open-ended writing prompt used to solicit learners’ reflections as part of the regular educational processes in the mentoring program studied. The prompt remained consistent, edited for clarity of instruction.*


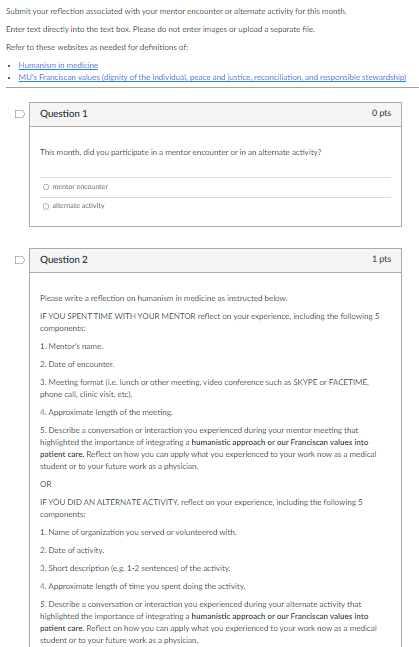


**Online Resource 3**

*This appendix describes details of the data collection and purposeful sampling method employed to maximize diversity within the data set.*

Assignments were securely stored in Canvas learning management system. For research purposes, we exported data from Canvas to Microsoft Excel, where each reflection was matched to learner attributes before being de-identified by a research assistant. Learner attributes included 1) program-related attributes (e.g., year in training, years in program) and 2) self-reported demographics (e.g., sex, religion). Before analysis, research assistants edited the data for readability (e.g. removing extra spaces), redacted identifiers, and ensured completeness. Data and demographics were loaded into qualitative analysis software, NVivo Version 14 (Lumivero, 2023). Students who did not consent to participate in the research were removed from the data before upload.

Our sampling approach included the following steps:

- We generated a sample to include all participants of low representation relative to the general data corpus (e.g., age over 29 or under-represented race in medicine).
- We added participants from religious minority groups (non-Catholic and non-Christian, given the program setting at a Catholic institution with Christianity highly represented among the student body). Because inclusion of all participants in certain religious minority groups created a data set much larger than we anticipated needing for saturation, we limited these subsamples by academic year, selecting to balance the dataset across all eight years. For example, Muslim students were highly represented in the data corpus, and the years 2018-2019 and 2022-2023 had relatively low representation in the sample, so we included all participants from just those two years who selected “Islam” for their religion.
- The race/ethnicity and religion demographic survey items included an ‘Other/please describe’ option where participants could write in their identity. Where five or more participants reported an identity that had not been offered for selection, we created additional demographic categories. Identities we grouped together are detailed in the caption of Table 1.

Having created the sample, we confirmed all identities were represented by at least five student-years each, where available. This decision was based on the assumption that five student-years of data from each of the minority attributes would generate a sample size likely to reach saturation.

Finally, we added a random sampling of five participants who selected all majority identities (i.e. five white, male Catholics aged 29 or younger). These identities were defined as ‘majority’ identities because none were included in the sampling method used to ensure inclusion of minority identities.

**Article Title:** Exploration of Osteopathic Medical Students’ Reflections on Humanism in a Values-Centered Mentoring Program

**Journal Name:** Medical Science Educator

**Authors:** Emily Young, MD, MEd, Naomi Schmalz, PhD, Khadijah Guisse, Linda Regan, MD, MEd

Correspondence should be addressed to Emily Young, Marian University Wood College of Osteopathic Medicine, 3200 Cold Spring Road, Indianapolis, IN; telephone: 317-955-6627; e-mail: [eyoung@marian.edu](mailto:eyoung@marian.edu).
